# High levels of multidrug resistant tuberculosis in new and treatment-failure patients from the Revised National Tuberculosis Control Programme in an urban metropolis (Mumbai) in Western India

**DOI:** 10.1186/1471-2458-9-211

**Published:** 2009-06-29

**Authors:** Desiree TB D'souza, Nerges F Mistry, Tina S Vira, Yatin Dholakia, Sven Hoffner, Geoffrey Pasvol, Mark Nicol, Robert J Wilkinson

**Affiliations:** 1 The Foundation for Medical Research, 84 – A. R. G. Thadani Marg, Worli, Mumbai 400 018, India; 2 Swedish Institute for Infectious Disease Control, Sweden; 3 Wellcome Centre for Clinical Tropical Medicine, Division of Medicine, Imperial College London, W2 1PG, UK; 4 Institute of Infectious Diseases and Molecular Medicine and Department of Medicine, Faculty of Health Sciences, University of Cape Town, South Africa; 5 National Institute for Medical Research, The Ridgeway, Mill Hill, London, NW7 1AA, UK

## Abstract

**Background:**

India, China and Russia account for more than 62% of multidrug resistant tuberculosis (MDRTB) globally. Within India, locations like urban metropolitan Mumbai with its burgeoning population and high incidence of TB are suspected to be a focus for MDRTB. However apart from sporadic surveys at watched sites in the country, there has been no systematic attempt by the Revised National Tuberculosis Control Programme (RNTCP) of India to determine the extent of MDRTB in Mumbai that could feed into national estimates. Drug susceptibility testing (DST) is not routinely performed as a part of programme policy and public health laboratory infrastructure, is limited and poorly equipped to cope with large scale testing.

**Methods:**

From April 2004 to January 2007 we determined the extent of drug resistance in 724 {493 newly diagnosed, previously untreated and 231 first line treatment failures (sputum-smear positive at the fifth month after commencement of therapy)} cases of pulmonary tuberculosis drawn from the RNTCP in four suboptimally performing municipal wards of Mumbai. The observations were obtained using a modified radiorespirometric Buddemeyer assay and validated by the Swedish Institute for Infectious Disease Control, Stockholm, a supranational reference laboratory. Data was analyzed utilizing SPSS 10.0 and Epi Info 2002.

**Results:**

This study undertaken for the first time in RNTCP outpatients in Mumbai reveals a high proportion of MDRTB strains in both previously untreated (24%) and treatment-failure cases (41%). Amongst new cases, resistance to 3 or 4 drug combinations (amplified drug resistance) including isoniazid (H) and rifampicin (R), was greater (20%) than resistance to H and R alone (4%) at any point in time during the study. The trend for monoresistance was similar in both groups remaining highest to H and lowest to R. External quality control revealed good agreement for H and R resistance (k = 0.77 and 0.76 respectively).

**Conclusion:**

Levels of MDRTB are much higher in both previously untreated and first line treatment-failure cases in the selected wards in Mumbai than those projected by national estimates. The finding of amplified drug resistance suggests the presence of a well entrenched MDRTB scenario. This study suggests that a wider set of surveillance sites are needed to obtain a more realistic view of the true MDRTB rates throughout the country. This would assist in the planning of an adequate response to the diagnosis and care of MDRTB.

## Background

India, designated as a 'high burden' country for tuberculosis (TB) has also been identified as a hot spot region for multi-drug-resistant *Mycobacterium tuberculosis *(MDRTB) infection [1,2]. Together India, China and Russia account for more than 62% of the global MDRTB burden [3]. Yet in the WHO Reports on global TB control, MDRTB in India continues to be reported between 2.5–2.8% and 14–17% amongst new TB and retreated patients respectively [4-8].

These reports are limited because of relatively small sample size, the absence of explicit criteria for patient selection, unclear definitions of retreatment, absence of quality control in laboratory methods and geographic restriction to sentinel sites (in which programme performance may exceed that of routinely monitored locations). Additionally the cases sampled from surveys in fixed sentinel sites in India approximate only 7% of the estimated MDRTB load of 110,132, in contrast to South Africa which has sampled almost 38% of its estimated burden of 14,034 MDRTB cases uniformly from different regions of the country [9,7]. The multiple challenges in measurement of the true burden of MDRTB are presented by Cohen et al 2007 [10]. They suggest that periodic surveys (the prime method for assessing levels of MDRTB in resource deficient settings) may underestimate total MDRTB burden because acquired drug resistant cases are undercounted and resistance amongst prevalent cases is not assessed.

Currently a 5 year (2006–2010) surveillance for drug resistance is being carried out in 10 states in India [11]. A preliminary report providing limited information from surveillance in 2 states concludes that the prevalence of MDRTB is ~3% in new cases and 12–18% amongst re-treatment cases. It furthermore concludes that there has been no increase in drug resistance over the past years as shown by studies from Tuberculosis Research Center, Chennai [11].

In contrast to these national and WHO reports, key studies from a private tertiary care facility in the TB hyperendemic metropolitan city of Mumbai in Western India in 2006 reported increasing trends of MDRTB in new and retreated cases [12]. Their report published in 2003 quotes MDRTB in new cases at 30% and in treated cases at 60% [13]. A recent report from the same centre highlighted the presence of 8% XDRTB in a subset of their MDRTB cases [14]. These figures generated through purposive sampling of patients accessing a tertiary care centre are critiqued by the national control programme as "unrepresentative". However despite being open to referral bias, they are clearly a cause for concern.

There are no community based data on MDRTB from an endemic setting such as Mumbai because drug susceptibility testing (DST) is not routinely carried out as a part of programme policy and the public health laboratory infrastructure is limited and ill equipped to cope with large scale testing. Only two intermediate reference laboratories have been established and accredited in the States of Maharashtra and Gujarat, whilst 11 other such State laboratories are still in the process of accreditation [15].

Our aim therefore, was to gain a community based estimate of the levels of drug resistance in previously untreated new and first line treatment-failure cases. This was undertaken in selected wards of Mumbai, a city vulnerable to drug-resistant disease because of its high population density, high prevalence of TB (299/100,000) [7] and an overstretched public, coupled with an unregulated private, health sectors [16]. Since conditions in Mumbai are likely to be markedly different from sentinel sites, it was considered likely that rates of MDRTB in Mumbai may differ substantially from published national estimates.

## Methods

### Location of study

The Revised National Tuberculosis Control Programme (RNTCP) in Mumbai is implemented in individual wards of the city. Since this study was part of a larger epidemiological project on transmission of MDRTB in an endemic setting, 4 centrally located wards F/N, G/N, H/E, and K/E characterized by a high sputum-positive case load, with moderately suboptimal cure rates ranging between 78–81% were selected. Fifty percent of the wards in Mumbai (11/23) displayed similar cure rates; an additional 20% of the wards even further reduced cure rates of 50–74% (RNTCP quarterly reports 2001). These 4 moderately suboptimal performing wards were selected to minimize the possibility of extreme outcomes due to selection bias. As far as could be ascertained there was no apparent deviation in the RNTCP functioning in these wards compared to the other wards in Mumbai. A significant proportion of the resident population of these 4 selected wards belonged either to the middle/lower socioeconomic class and resided in informal housing in slums and were therefore thought to be representative. Cumulatively the 4 wards comprised of 38 DOTS Centers, covering a population of 3 million. Each field worker covered an average of 9 DOTS Centers on a thrice weekly basis.

### Calculation of sample size

Sample size was based on an earlier study which reported 30% and 60% MDRTB in new and retreated cases respectively from the same setting albeit from a tertiary care centre treating outpatients [13]. Thus based on an absolute error of 8% at 95% confidence, the sample size for previously untreated cases and first line treatment-failures [viz. sputum-smear positive at the fifth month after commencement of a 2(HERZ)_3 _+ 4(HR)_3 _regimen comprising of 2 months of HERZ thrice weekly followed by 4 months of HR thrice weekly] was estimated to be 224 and 47 respectively.

### Patient classification and recruitment

Since the patient sample was drawn from suboptimally performing wards, the procedure for screening and recruitment of patients was designed to project balanced figures for MDRTB levels and minimize bias caused by previous TB treatment and interruption in treatment of more than 2 weeks.

**Figure 1:**
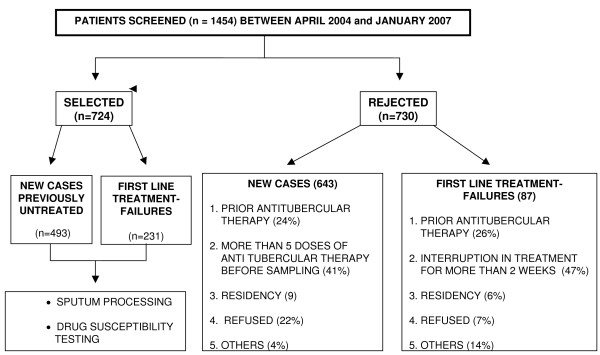
**Study design**.

During the study period from April 2004 to January 2007, two groups of sputum smear-positive pulmonary TB patients registered with the RNTCP DOTS (Directly Observed Therapy Short Course) Centers were identified for inclusion. These consisted of – i) newly diagnosed, previously untreated patients at onset of therapy and ii) first line treatment-failures {viz. sputum positive at the fifth month after commencement of a 2(HERZ)_3 _+ 4(HR)_3 _regimen} (Figure 1). Field workers scrutinized treatment cards and District TB registers. In keeping with the larger study objective of investigating MDRTB transmission, the selection of as many new cases as was logistically feasible at a DOTS center was attempted subsequent to the confirmed presence of a first line treatment-failure at the same Center. Furthermore screening was based solely on patient availability and contact at the health posts. Logistical feasibility was influenced by factors such as the low ratio of field workers to health posts, extreme climatic conditions compounding difficult transport logistics and delays in systemic documentation of new patients in registers.

Inclusion criteria for patients included i) smear-positivity, ii) age 15–70 years (to exclude those patients with a non-productive cough), iii) residency in Mumbai for at least 3 years immediately prior to diagnosis and (iv) residency in the same area as the health posts where treatment was sought. Patients with a definitive past history of TB or antitubercular therapy prior to the existing episode, as determined through a schedule and/or scrutiny of district TB registers and patients who interrupted treatment for more than 2 weeks (in case of first line treatment-failures) were excluded to minimize bias towards MDRTB detection [17]. As far as could be ascertained, patients in the treatment-failure group had not received any antitubercular therapy prior to the current episode.

Besides preliminary scrutiny of treatment cards and District TB registers to identify the 2 groups of patients (untreated and treatment-failures), screening was directed through a standard schedule to i) detect past history of TB/antitubercular treatment, ii) determine period of residency in Mumbai and iii) detect occurrence of treatment interruption in treatment-failures. Patients were recruited after informed consent and referred to Voluntary Counseling and Testing Centers for HIV counseling and testing.

Clearance for this study was obtained from the Foundation for Medical Research (FMR) Institutional Ethics Committee (20.07.2001/01).

### Drug susceptibility testing

Early morning sputum samples from patients were processed by the modified Petroff's method [18], stained by Ziehl-Neelsen Carbol Fuchsin, microscopically examined and cultured on Lowenstein-Jensen slopes (Himedia, India) as well as in Dubos broth (Himedia, India). Culture-negative or contaminated samples (~3%) were excluded from the analysis. Biochemical tests for niacin and catalase production were performed to confirm the identity of *Mycobacterium tuberculosis*. Drug susceptibility testing of the samples was performed by the radiorespirometric Buddemeyer technique (a manual modification of the Bactec 460 technique) [19,20]. Briefly, samples were inoculated into Dubos broth containing ^14^C Palmitic acid (Board of Radiation and Isotope Technology, India). Vials were set up in triplicate each containing 0.5 × 10^6^/ml of Acid Fast Bacilli (AFBs) in absence (positive control) as well as presence of drugs (μgs/ml): Isoniazid (H – 0.1), Rifampicin (R – 2), Pyrazinamide (Z – 100) and Ethambutol (E – 2.5). Negative controls consisted of medium without acid fast bacilli (AFBs) as well as with heat killed AFBs. A 1:100 dilution of the positive control was also maintained. Readings were obtained daily until the eighth day in counts per minute (cpm) on a Wallac 1409 DSA liquid scintillation counter. Growth indices (GI) were calculated for the drug containing vials and the 1:100 positive control. Difference in growth indices (ΔGI), identical to that applied in the Bactec 460 method, calculated over consecutive days was used to determine susceptibility. The value of the mean ΔGI in the triplicate drug containing vials was compared to that for 1:100 control for the same day. If ΔGI was less in the drug containing vials than the 1:100 control, the bacteria were considered susceptible; if more, they were considered resistant [21,22].

Multidrug resistance (MDR) was defined as resistance to at least H and R. Other cases were categorized as follows: **Drug sensitive **– absence of resistance to any of the drugs, **monoresistance **– resistance to only 1 drug and **polyresistance **– resistance to at least two or more drugs excluding the HR combination.

### Quality Control

#### External and Internal Validity

Ten percent of the clinical mycobacterial isolates were sent single-blinded to the Swedish Institute for Infectious Disease Control, Stockholm, a WHO/IUATLD supranational reference laboratory for external quality assurance of the DST of the *Mycobacterium tuberculosis *isolates by the Bactec 460 method. Isolates were also retested in our laboratory to determine reproducibility of the results. The isolates were subcultured in Dubos medium for 4 weeks before retesting. For discordant drug susceptibility results between the two laboratories, the results of the supranational referral laboratory have been reported whilst for internally discordant results, the initial results have been presented.

### Statistical analysis

The data was analyzed utilizing SPSS version 10.0 and Epi Info 2002 using Chi square tests. An age group stratification of 15–35 yrs, 36–55 yrs and 56–69 yrs was applied for purposes of analysis. The agreement of DST between our laboratory and the Swedish Institute for Infectious Disease Control, Stockholm, was ascertained through generation of kappa values individually for the four first line drugs [23]. The kappa values were interpreted as follows: ≤ 0.40 = Poor, 0.4 – 0.75 = Good, > 0.75 = Excellent. Comparisons were further expressed in terms of 'sensitivity' and 'specificity' for the four drugs using standard definitions.

## Results

### Patient selection

A total of 1,454 patients (1,136 previously untreated and 318 first line treatment-failures) were screened at the health posts between April 2004 and January 2007. Of the 2,184 smear positive cases presenting to the RNTCP for diagnosis, 1,136 (52%) were screened due to reasons outlined previously.

Finally, based on the inclusion criteria described earlier, 724 cases comprising of 493 previously untreated and 231 first line treatment-failure cases were recruited. A similar number namely 730 cases (643 previously untreated and 87 first line treatment-failures) had to be excluded. The various reasons for exclusion were prior antitubercular treatment for more than 1 month, residency outside Mumbai, accessing the private sector in preference to the RNTCP and transfer to a different DOTS center or interruption in treatment for more than 2 weeks (only in case of treatment-failures) (Figure 1).

### Patient characteristics

Of the 724 recruited cases, the majority belonged to the 15–35 yrs age group; 73% (359/493) among previously untreated cases and 66% (153/231) among first line treatment-failures. The majority were male with no difference in gender distribution between the previously untreated cases – 301/493 (61%) and first line treatment-failures – 139/231 (60%). The HIV positivity rate was also comparable at 5% and 3% respectively in the 2 groups (p = 0.105).

### Drug susceptibility testing

#### Newly diagnosed, previously untreated

A high level of drug resistance was seen amongst the previously untreated cases. Amongst the MDR patients (24%), the proportion of resistance to 3 or more drugs including HR (20%) was greater than that of resistance to HR only (4%) (Table 1). Monoresistance was highest to H at 11% (53/493) and lowest to R at 1% (3/493) with 5% resistant to Z (27/493). No association between drug susceptibility and age could be determined by univariate analysis.

**Table 1:** Drug susceptibility profiles in TB patient groups

GROUP	SENSITIVE	RESISTANT				
		
		MONO	a) HR	b) HR + E/Z/EZ	MDR (a+b)	POLY
First time treatment-failures (n = 231)	39 (17)	38 (16)	13 (6)	82 (35)	95 (41)	59 (26)
Newly diagnosed (previously untreated) (n = 493)	175 (35)	103 (21)	19 (4)	98 (20)	117 (24)	98 (20)

(Key: H: Isoniazid, R: Rifampicin, Z: Pyrazinamide, E: Ethambutol,

Figures in parentheses are %,

Polyresistance: Resistance to ≥ 2 drugs exclusive of the HR combination)

**Figure 2:**
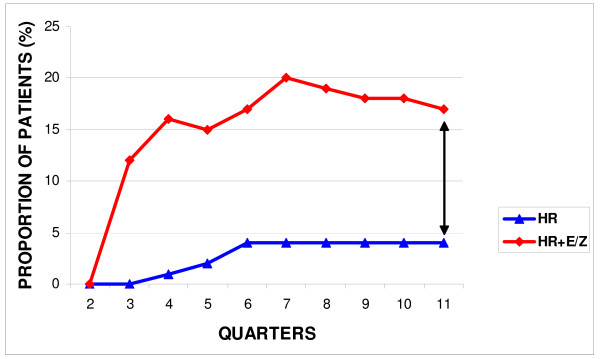
**Cumulative proportionate increase in drug resistance in new cases**.

Strikingly, at all 11 three monthly intervals of the study, the cumulative proportion of previously untreated cases resistant to 3 or more drugs inclusive of HR was higher than that resistant to HR alone (Figure 2).

#### First line treatment-failures

As expected, a high proportion of MDRTB and polyresistance, 41% (95/231) and 26% (59/231) respectively, was observed amongst the first line treatment-failures. A significantly higher proportion of failures who were MDR were in the age group of 36–55 years as compared to those whose strains were sensitive/monoresistant (p < 0.022). A significantly higher number of treatment-failures were MDR as compared to the new cases (p = 0.0). The monoresistance profile for the 4 drugs tested was similar to that seen in previously untreated cases remaining highest to H at 7% (17/231) and lowest to R at 2% (4/231), 4% showing resistance to Z (10/231). No association was detected between the drug susceptibility profiles of the strains and the HIV status of the patients within either group.

#### Quality Control

Kappa scores showed excellent agreement (range 0.76–0.77), between the drug sensitivity results obtained at FMR and by the supranational referral laboratory for H and R. Sensitivity and specificity for the four individual drugs ranged from 80– 100%, and 87–94% respectively. As reported elsewhere [24], there was greater discordance for observations relating to E between the two centers as expressed by the kappa values and the sensitivity estimate. Internal comparison also indicated a high degree of reproducibility for DST (Table 2).

**Table 2:** External and Internal validity of drug susceptibility testing for individual first line drugs

	**EXTERNAL^a ^(n = 45)**				**INTERNAL^b ^(n = 70)**	
**Drugs**	**Concordance** **(%)**	**Kappa Values**	**Sensitivity** **(%)**	**Specificity** **(%)**	**Concordance** **(%)**	**Kappa Values**

H	91	0.773	86	94	95	0.893
R	93	0.762	100	93	97	0.919
Z	87	0.557	100	87	91	0.819
E	89	0.487	80	91	90	0.759

(Key – a: Performed at Swedish Institute for Infectious Disease Control, Stockholm,

b: Repeated at FMR,

H: Isoniazid, R: Rifampicin, Z: Pyrazinamide, E: Ethambutol)

## Discussion

The problem of MDRTB in Mumbai has been previously highlighted [25]. A high prevalence of MDRTB strains (11–68%) was reported in hospital based studies by 2 laboratories between 1991 and 2006 [12,26] and the threats of its transmission, stressed [27]. Unfortunately as stated previously such data continues to be overlooked. This study, with its emphasis on screening of RNTCP outpatients from defined communities, careful selection criteria for previously untreated and treated patients and corroboration via external quality assurance was designed to provide a view of drug resistant TB in programmatically vulnerable areas of this urban metropolis.

Inherent in the study design are three points at which bias could have been introduced thereby leading to potential under- or over-estimate of the prevalence of MDRTB in previously untreated cases. Firstly, only 52% of the cases registered with the RNTCP were screened due to reasons mentioned in the results. Of the 48% who did not enter the study, it is likely that a proportion would have been excluded on the evidence of previous treatment. However it is equally possible that the enlarged number may have resulted in a lower rate of MDRTB than that reported here. Thus the possibility of bias generated towards MDR by the reduced intake prior to screening remains open. Secondly, despite our efforts at excluding previously treated patients during screening, it is possible that those included for DST may not have admitted to taking prior treatment. This is a likely cause for an overestimation of MDRTB in new cases. Thirdly, of the 1,136 screened patients registered as new cases, 24% had to be excluded because they had some sort of history of TB treatment. An additional 41% were excluded since they had consumed more than 5 doses of anti tubercular therapy prior to sampling (Figure 1), to reduce bias towards MDRTB. Whilst this could tend towards an underestimate in the total MDRTB prevalence in the area, there could have been other selection pressures that we were unable to capture in the exclusion algorithm. An important example would be a history of contact with a patient at increased risk of MDRTB (e.g. a treatment-failure case). We did attempt in the screening to establish connections of new cases to TB patients especially since new patients and treatment-failures accessing a health post are drawn from the same defined geographical area which it is supposed to serve. However patients would be unlikely to be able to articulate regarding MDRTB in their family or vicinity.

Acknowledging the bias generated in the incorporation of such vulnerable wards, the emphasis in patient selection was a concerted attempt to estimate the MDRTB levels in previously untreated cases. This was expected to offset the drug resistance in previously treated cases that often present to the health system as new cases. Despite this, this study has documented high levels of multiple drug resistance (both MDR and polyresistance) amongst previously untreated cases in the urban wards. Rifampicin resistance has been reported to be a sentinel marker for MDR [28]. Moreover 96% of the mutations associated with rifampicin resistance lie within the rpoβ region [29]. Hence over and above the confidence given by external quality assurance, genotypic corroboration of R resistance in a subset of previously untreated and first line treatment-failure cases was also obtained by the InnoLiPA line probe assay which screens for mutations in the rpoβ gene [29] (88% agreement, k = 0.74, data not shown).

Our initial MDRTB figures differ greatly from those reported by the RNTCP and Indian national figures in WHO global surveys [4-8,30,31]. The level of MDRTB in first episode treatment failures in our study whilst less than that reported by Rodrigues et al, 2006 (41% vs. 68%), is higher than the 17% documented by Santha et al, 2005 and Chauhan, 2008. This comparison is made difficult since definitions of treatment-failures in these publications are not precise. Nevertheless our figures remain a cause for concern for several reasons which have been reported earlier [32] and are outlined below.

In view of the high levels of MDRTB amongst previously untreated patients, it is possible that a proportion of first line treatment-failures may have had initial drug resistance, which was undetected due to lack of DST facilities. Observations in a subgroup of patients whose MDRTB status at diagnosis and post treatment was tested in our laboratory and whose treatment outcomes were recorded, revealed that 13% (21/162) of the failures were MDR at onset (data not shown). Alternatively, detection of MDR at the fifth month could be due to acquisition of resistance by an initially sensitive strain or a further exogenous drug resistant strain contracted during the treatment period [33,34].

Whilst this study was not primarily intended to determine the prevalence of MDRTB, the linking of MDR in the treatment-failures to population-based treatment outcome rates is difficult to establish in this situation. Observations from treatment registers for the same cohort revealed a 27% drop out rate of patients who were not traceable or who had defaulted during the course of their first line treatment (data not shown). Such patients are in all likelihood omitted from the denominator when cure rates are computed. This may be further compounded by the lack of external quality assurance in sputum-smear microscopy. Moreover there have not been any formal external evaluations of the validity of reported cure rates in the national programme [35].

Whilst our observations may not be generalizable to the entire country or even well performing wards of Mumbai, observations from poor programme performance areas may serve to highlight factors implicated in the development of drug resistance [36]. These include the continued use of intermittent therapy (discontinued by WHO since 2004), suboptimal levels of drugs in patients due to erroneous pattern of drug intake or drug side effects such as vomiting and above all, the unavailability of DST facilities among treatment-failures after first line and retreatment regimens. Such patients will continue to transmit MDRTB even after being readmitted to the RNTCP where Category 2 treatment {3(SHERZ)_3 _+ 5(HRE)_3_} (with the addition of only a single drug streptomycin) maybe suboptimal [37] and where provision of diagnosis and treatment of drug resistant TB is currently unavailable or unaffordable.

Whilst many patients from our study wards are likely to access the RNTCP for TB treatment, the seeking of care from private practitioners by many others and the constant shopping for treatment between the public and private sectors cannot be ignored. The contribution to MDRTB levels from aberrant treatment practices in the unregulated private sector has been suspected for long and makes it likely that the levels of MDRTB revealed by this study are an underestimate [38].

A major concern and one of great biological interest, is the high level of resistance to 3 or even 4 first line drug combinations in comparison to HR alone (Table 1). Additionally, the proportion of previously untreated cases resistant to more than 3 drugs inclusive of HR was higher than that resistant to HR alone throughout the study period (Figure 2). This scenario of amplified drug resistance suggests the presence in this setting of a protracted, well entrenched, evolving MDR scenario [1]. Preliminary evidence from 14 MDRTB isolates from our cohort does not reveal the presence of extensively drug resistant strains (XDR defined as MDR strains additionally resistant to an injectable aminoglycoside and any of the fluoroquinolones) (data not shown). However the potential threat of XDRTB strains in the light of several systemic and infrastructure limitations [36,38] should not be underestimated since their emergence within Mumbai city and elsewhere in the country has already been documented [14,39].

Unlike the scenario in South Africa [40] and Russia [41], the high proportion of MDRTB in Mumbai does not appear to be greatly influenced by HIV infection, as the seropositivity amongst our MDRTB cases (range 3–5%) was not overtly different from the national prevalence rates of 5% [42].

Preliminary fingerprinting studies using spoligotyping on the same cohort of patients reveals a striking amount of hetereogeneity in strains and overall lack of an association between large clusters and MDRTB. The proportion of Beijing, a strain known to be globally associated with MDRTB epidemics is only 4% in the overall cohort in Mumbai and virtually absent in the rural areas in proximity to the metropolis. (Chatterjee et al, submitted for publication). Cumulatively, this argues against the occurrence of a single drug resistant strain epidemic in Mumbai.

A gradual realization of the significant presence of MDRTB within the country is apparent from the introduction of DST for first line and retreatment failures, and provision for DOTS Plus in the RNTCP since 2007 in certain selected areas in India. However, despite the selectivity in study design, if initial MDRTB is high as suggested by this and other studies [12,26], then a window for transmission of MDRTB would remain open for 5–6 months in case of new (first line treatment) and 8 months for retreated (Category 2) patients.

## Conclusion

Our findings strongly suggest that national estimates have been unable to capture locale-specific variations in MDRTB in the country largely because they originate from sentinel sites where programme operational factors are likely to be optimal. The data highlights systemic and environmental factors that have been implicated in the development of drug resistance in operationally weak areas (such as the selected wards) and the need for drug resistant TB surveillance to be incrementally achieved, through regular systematic studies that randomly sample TB patients [43] and supplement sputum microscopy with ranked risk evaluations and/or DST (Atre et al, submitted for publication). This would entail i) the establishment of quality laboratory infrastructure capable of undertaking reliable DST in a wider set of surveillance sites that can reflect a more balanced view of the ground realities in vulnerable locations, ii) choice for appropriate technology for DST and iii) judicious introduction of DOTS Plus combined with measures of care for patients with drug resistant TB.

## Competing interests

No author has a financial or other conflicts of interest related to this work. None of the authors have an association that poses any conflict of interest. The funders had no part in the decision to publish the manuscript.

## Authors' contributions

DTBD was the study coordinator responsible additionally for the standardization and quality assurance of the radiorespirometric technique for drug sensitivity testing and analysis of results. NFM was Principal Investigator (India) of the Project responsible for the study design and analysis of results. TSV was responsible for the routine drug sensitivity testing, reporting of results and analysis. YD was the chest physician who undertook selection of patients in the study based on clinical criteria and also had a role in operations and field aspects of patient recruitment and liaisoning with Mumbai District TB Control Society. SH undertook external quality assurance testing for drug susceptibility. GP was the Principal investigator (U.K.) of the Project who helped in formatting the presentation of data and reinforcing of issues in the discussion. MN assisted with continuous inputs into data analysis and interpretation and extensive editing of the manuscript. RJW assisted with continuous inputs into study design, data analysis and interpretation and extensive editing of the manuscript. All authors were involved in the drafting of the manuscript and have read and approved the final version.

## Pre-publication history

The pre-publication history for this paper can be accessed here:


